# Supplementary material for: High mortality rates and long-term complications in children with infectious brainstem encephalitis: A study of sixteen cases
Source: PLoS One. 2025 Feb 4;20(2):e0318818. doi: 10.1371/journal.pone.0318818 (PMC11793775; doi:10.1371/journal.pone.0318818)
Supplement: Attachment — Submitted filename: Response to Reviewers.docx [file pone.0318818.s001.docx]

Author’s response to reviewers

Reviewer #1:

A retrospective study was conducted on pediatric brainstem encephalitis (BE) cases at a National Children's Medical Center from 2015 to 2024.

I would expect the following from a retrospective study:

1. BE is a rare disease, and I would expect an analysis of its frequency, demographic patterns, and risk factors over a long period.

Author’s response:

Thank you for your valuable suggestions. Infective BE in children is indeed a rare clinical condition, yet its severity necessitates heightened vigilance among clinicians. To date, no incidence data could be found in the literatures. Given that patients at our Children's National Medical Center are typically complex or critically ill, this patient population may not accurately reflect the overall prevalence of infectious BE. In this paper, we provide annual case counts to illustrate the potential frequency of this disease. Specifically, among 3 million children visiting the outpatient department and 500,000 hospitalized children, there were 16 and 13 cases, respectively. In the revised manuscript, we have integrated these two sets of data for a comprehensive presentation. And we have incorporated Table 1 into the revised draft to offer a comprehensive overview of the clinical information pertaining to these 16 cases.

In response to the reviewer's suggestion, we have incorporated the age range into the demographic characteristics section of the revised manuscript. Concerning the description of risk factors, given that only one out of the 16 patients had an underlying condition (congenital heart disease), demographic variables such as age and sex did not appear to be indicative of risk factors, likely due to the limited sample size. In the discussion section, we have provided additional clarification regarding the risk factors.

2. I also expect clinical manifestations and diagnosis to highlight common symptoms and pathogens causing BE, providing clues for diagnostic protocols and management.

In this study, the authors reported key symptoms such as fever, consciousness disorders, and seizures. However, I suggest including additional data such as demographic details, personal history (e.g., autoimmunity, immune checkpoint inhibitor exposure), immune response markers, brainstem-specific signs and symptoms for a more detailed analysis. A risk factor analysis should also be included.

The authors also reported the pathogens associated with BE, cranial imaging, and electroencephalogram findings, as well as the outcomes of several treatments. However, the data could be more comprehensive by including MRI findings, CSF analysis, oncological features, and antibody-specific associations. While the authors mentioned that CSF data is unavailable, its inclusion would greatly enhance the study's value.

Overall, I consider this study preliminary. More data could be added as suggested above, along with deeper analyses. The illustrations can also be improved.

Author’s response:

We would like to express our gratitude to the reviewers for acknowledging the clinical and scientific significance of the manuscript, as well as for their insightful recommendations. In response, we have augmented Table 1 with detailed clinical information pertaining to the 16 patients, including cerebrospinal fluid test results.

Reviewer #2:

Brainstem encephalitis is defined as a disorder characterized by myoclonus, ataxia, nystagmus, kinetic nerve palsy, and bulbar palsy and various combinations of these disorders, whether or not confirmed by neuroimaging. They are often easily overlooked by physicians and parents, and this may lead to misdiagnosis. If the medullary vital centers are involved, the condition can deteriorate rapidly and even lead to death. Early diagnosis of the presence of a viral infection and early intervention are critical.

The paper analyzes the prevalence of brainstem encephalitis among 30 million outpatients and 500,000 inpatients from real-world clinical data over the past decade, and analyzes the etiology, clinical manifestations, imaging, diagnosis and treatment, and prognostic follow-up in detail.

The paper is concise, logical, strongly argued, with accurate data and clear conclusions.

For clinicians, researchers and patients, it is a rare first-hand data, which is of great reference value. Especially for this kind of disease with atypical symptoms, easy to misdiagnose, low population prevalence relative to common diseases, and poor prognosis once the disease develops, timely and adequate retrospective study is very necessary. This is also reflected in the analysis of infectious etiologies, including, but not limited to, enteroviral involvement in neurological infections, etc., which are well worth following up with studies of pathogenic mechanisms to facilitate the development of effective drugs and vaccines.

Finally, I would like to make a few suggestions without prejudice to the evaluation of the full paper.

The baseline characteristics of patients (Line 105), clinical manifestations (Line 126), etiology (Line 139), prognosis (Line 177), and follow-up (Line 192) should be matched with corresponding figures and tables, rather than just textual descriptions, which may be more convincing and convenient for the readers to understand the data information intuitively, especially the baseline characteristics of the cohort and the survival curve.

Author’s response:

Thank the reviewers for their insightful and professional comments. Given the rapid progression, serious consequences, and lack of clinical specificity associated with infectious brainstem encephalitis, timely diagnosis often necessitates a combination of clinical vigilance and laboratory examinations, including neuroimaging, to facilitate prompt and targeted treatment measures, thereby preventing adverse outcomes. Based on the reviewers' suggestions, we have supplemented the detailed information of these 16 patients in a more intuitive format as presented in Table 1. We anticipate that the successful publication of our manuscript will contribute to a deeper understanding of infectious BE among clinicians and benefit patient care.
